# TANK-Binding Kinase 1-Dependent Responses in Health and Autoimmunity

**DOI:** 10.3389/fimmu.2018.00434

**Published:** 2018-03-06

**Authors:** Cynthia Louis, Chris Burns, Ian Wicks

**Affiliations:** ^1^Inflammation Division, The Walter and Eliza Hall Institute of Medical Research, Parkville, VIC, Australia; ^2^Chemical Biology Division, The Walter and Eliza Hall Institute of Medical Research, Parkville, VIC, Australia; ^3^Rheumatology Unit, Royal Melbourne Hospital, University of Melbourne, Parkville, VIC, Australia

**Keywords:** TANK-binding kinase 1, type 1 interferons, germinal center, autophagy, humoral immunity, autoimmunity

## Abstract

The pathogenesis of autoimmune diseases, such as rheumatoid arthritis (RA) and systemic lupus erythematosus (SLE) is driven by genetic predisposition and environmental triggers that lead to dysregulated immune responses. These include the generation of pathogenic autoantibodies and aberrant production of inflammatory cytokines. Current therapies for RA and other autoimmune diseases reduce inflammation by targeting inflammatory mediators, most of which are innate response cytokines, resulting in generalized immunosuppression. Overall, this strategy has been very successful, but not all patients respond, responses can diminish over time and numerous side effects can occur. Therapies that target the germinal center (GC) reaction and/or antibody-secreting plasma cells (PC) potentially provide a novel approach. TANK-binding kinase 1 (TBK1) is an IKK-related serine/threonine kinase best characterized for its involvement in innate antiviral responses through the induction of type I interferons. TBK1 is also gaining attention for its roles in humoral immune responses. In this review, we discuss the role of TBK1 in immunological pathways involved in the development and maintenance of antibody responses, with particular emphasis on its potential relevance in the pathogenesis of humoral autoimmunity. First, we review the role of TBK1 in the induction of type I IFNs. Second, we highlight how TBK1 mediates inducible T cell co-stimulator signaling to the GC T follicular B helper population. Third, we discuss emerging evidence on the contribution of TBK1 to autophagic pathways and the potential implications for immune cell function. Finally, we discuss the therapeutic potential of TBK1 inhibition in autoimmunity.

## Introduction

TANK-binding kinase 1 (TBK1) is an IKK-related serine/threonine kinase best known for the induction of antiviral type I interferons (IFN-Is) in innate immunity. However, a growing body of evidence highlights the relevance of TBK1 for other responses. In this review, we discuss our present understanding of the role of TBK1 in nucleic acid sensing pathways, antibody responses, and autophagy. We conclude by speculating how these diverse TBK1-regulated responses could potentially culminate in the induction, promotion, and maintenance of autoimmunity, as well as how pharmacological modulation of TBK1 could represent an alternative treatment strategy, particularly in the context of humorally mediated autoimmunity.

### TBK1 Overview

**Figure 1:**
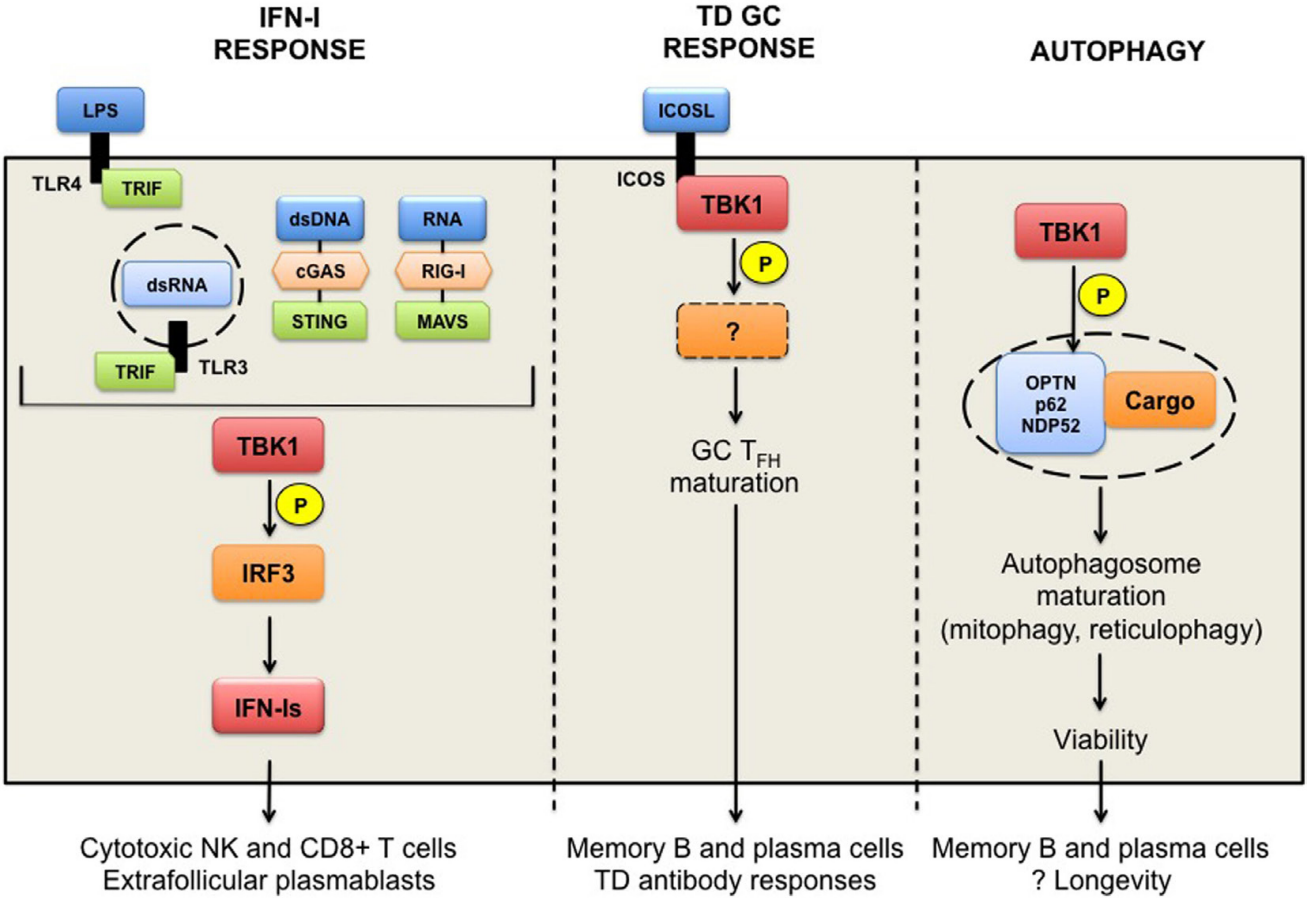
TANK-binding kinase 1 (TBK1) in humoral responses. TBK1 functions downstream of TLR3/4-TRIF and DNA receptor cGAS-STING pathways leading to the activation of the transcription factor interferon regulatory factor 3 and the production of interferons (IFN-Is). Chronic IFN-Is prime cytotoxic functions promote the survival of NK and CD8^+^ T cells, presumed to have pathogenic roles in autoimmunity, as well as the formation of extrafollicular plasmablasts. TBK1 is also implicated in the inducible T cell co-stimulator (ICOS) signaling pathway in T follicular B helper (T_FH_) cells to thymus-dependent (TD) antigens. TBK1 is recruited to and activated upon ICOS engagement to ICOS ligand, and promotes the maturation of pre-T_FH_ to germinal center (GC) T_FH_ cells. TBK1 targets downstream of ICOS signaling remain to be determined. TBK1-driven ICOS signaling is necessary for the generation of GC-derived memory B and plasma cells, and TD antibody responses. Finally, TBK1 can promote autophagy through the phosphorylation of autophagy receptors proteins (optineurin, p62, or NDP52), which sequester ubiquitinated cargo (damaged or redundant organelles). Mitophagy in memory B cells and reticulophagy in plasma cells are required for their longevity *in vivo*. TBK1 involvement in these processes remains to be determined.

TANK-binding kinase 1 is an IKK-related serine/threonine kinase, best known for the induction of innate antiviral type I IFNs. However, TBK1 potentially has much broader functions, which we discuss in this review (Figure 1). TBK1 is ubiquitously expressed in both hematopoietic and non-hematopoietic compartments. Germline deletion of TBK1 is embryonically lethal in mice (1), highlighting its homeostatic functions during development. Through biochemical studies, TBK1 was shown to be activated by double stranded (ds)-RNA (*via* TLR3-TRIF), LPS (*via* TLR4-TRIF), viral RNA (*via* RIG-I-MAVS), and dsDNA (*via* cGAS-STING) in innate immune signaling pathways (2, 3). TRIF (TIR-domain-containing adapter-inducing IFN β), MAVS (mitochondrial antiviral-signaling), and STING (stimulator of IFN genes) are innate immune adaptor proteins that transduce signal downstream of their corresponding sensors to the activation of interferon regulatory factor 3 (IRF3). Mechanistically, TBK1 activation is thought to occur *via* trans-autoactivation, in response to adaptor proteins that shuttle TBK1 to specific signaling complexes and direct subcellular localizations, such as to the ER-Golgi compartments (4–7). Activated TBK1 then phosphorylates IRF3 and induces the production of type I IFN-Is (8–12). Other TBK1 substrates include AKT (13, 14) and PLK1, which are involved in TLR activation or oncogenicity of cancer cells (15). Closely related to TBK1, IKKε shares 60% homology and is initially thought to participate also in IFN-Is induction (8, 9). Subsequent studies show that IKKε is dispensable for IFN-I responses (16). IKKε is abundantly expressed in T cells and have been shown to regulate a number of T cell responses (17–19).

Interferons are a family of cytokines with potent antiviral priming effects, but are also associated with humorally mediated autoimmune diseases, most notably systemic lupus erythematosus (SLE) (20–22). Recently, TBK1 was also shown to associate with the inducible T cell co-stimulator (ICOS) in CD4^+^ T follicular B helper (T_FH_) cells that support efficient antibody responses (23). However, the downstream target(s) of TBK1 in ICOS signaling have not yet been identified. Finally, TBK1 is also implicated in promoting autophagy by phosphorylating autophagy receptor proteins, including optineurin (OPTN), SQSTM1/p62, and NDP52 (24, 25). TBK1-mediated regulation of autophagy is currently under evaluation because TBK1 haploinsufficiency is a major risk factor in neurodegenerative diseases (26, 27). Autophagy is thought to protect senescent neuronal cells from the accumulation of defective or redundant organelles. TBK1 may likewise physiologically protect long-lived immune cells through autophagy (28). Although no particular TBK1 genetic variants have to date been directly linked to the development of autoimmune diseases, the diverse functions of TBK1 may contribute to one or more aspects of autoimmunity, which is the focus of this review.

### TBK1 and Type I IFNs

TANK-binding kinase 1 has a well described role in activating the transcription factor IRF3 to induce type I IFNs production (8–12). IFN-Is are a family of cytokines with pleiotropic functions that have potent antiviral and antimicrobial effects against some intracellular bacteria, but are also implicated in pathogenesis of SLE (SLE, discussed below). TBK1 is ubiquitously expressed in both hematopoietic and non-hematopoietic compartments and it is activated by sensor-adaptor pairs, including TLR3/4-TRIF, RIG-I-MAVS, or cGAS-STING, in response to LPS, dsRNA, virus infection, and cytoplasmic DNA, respectively (2, 3). Consequently, TBK1^−/−^ mouse embryonic fibroblasts (MEFs) have impaired production of IFN-Is (IFN-α and IFN-β) and IFN-inducible chemokines (CCL5 and CXCL10), among other genes, following activation with synthetic dsRNA (poly I:C) or viruses, or LPS (8, 10).

Molecular characterization of the sequence of events leading to TBK1 induction, IRF3 activation, and IFN-Is production have mainly been performed in cell lines and MEFs, in response to LPS stimulation or in the context of antiviral responses (2, 3, 8, 10). Elucidating roles for TBK1 in more complex biological settings *in vivo* has been challenging due to the embryonic lethality of germline TBK1-deficiency in mice. This is thought to be due to TNF-α-induced hepatocyte apoptosis and can be rescued by combined loss of TNF (i.e., TBK1^−/−^ TNF^−/−^ mice are viable) (1). Subsequently, TBK1 has been suggested to control cell survival through PAI-2/serpinB2 and transglutaminase 2 in the TNF-activated anti-apoptotic response (29).

High levels IFN-α or induction of IFN-stimulated genes (i.e., the “IFN signature”) is a remarkably consistent feature of SLE and is associated with high titers of affinity-matured autoantibodies and worse disease outcome (20, 21, 22). A similar IFN signature and correlation with high levels of autoantibodies and disease activity is also found in some patients with RA and primary Sjogren’s syndrome (30, 31) consistent with a pathogenic role for IFN-α in autoimmunity. Consequently, the possibility of targeting TBK1-dependent IFN-Is induction has received attention as a treatment strategy (32).

#### IFN-Is in Protective and Pathogenic Immune Responses

Among members of the IFN-I family in humans and mice, IFN-α and IFN-β are the best characterized and most broadly expressed. They signal through a shared, ubiquitously expressed heterodimeric receptor (IFNAR), and prime a rapid antiviral response that acts directly or indirectly on many cell types, including NK cells, T cells, B cells, DCs, and macrophages (33–35). IFNAR signaling mediates early attrition of existing memory CD8^+^ T cells in response to viral infections, which is thought to permit a more vigorous, diverse, and efficient T cell response emanating from the naïve T cell pool (36). In later stages, IFNAR signaling in activated cytotoxic CD8^+^ T cells (CTLs) (37) and NK cells (38) is important for long-term survival against perforin-mediated cytotoxicity, thereby preventing rapid elimination *in vivo* and sustaining antiviral immunity. IFNAR signaling is also required for optimal NK cell effector function through upregulation of granzyme B (38). IFN-Is is gaining attention in anti-cancer therapy, where it is generally considered pro-cytotoxic for CTLs and presumably NK cells. This is exemplified by the observations that IFNAR downregulation in CTLs endows colorectal cancers with an immune-privileged niche that promotes aggressive tumorigenesis, associated with poor prognosis, and lessens the response to immunotherapy. Conversely, IFNAR expression suppresses tumor growth and improves the efficacy of combined anti-cancer chimeric antigen receptor T cell transfer and PD-1 inhibition (39). Targeted intratumoral delivery of IFN-I-inducing (i.e., interferogenic) cyclic dinucleotide GMP-adenosine monophosphate (AMP), which activates the STING-TBK1 pathway and IFN-Is production in endothelial cells, has been shown to control tumor growth by boosting antitumor CD8^+^ T responses in murine models of melanoma and colon cancer (40).

Emerging evidence also implicates dysregulated NK cells and CD8^+^ T cells in SLE and potentially RA (41). Despite an overall reduction in circulating NK cell number in lupus patients and lupus mouse models, presumably owing to activation-induced death of these cells, NK cells with an activated phenotype infiltrate the kidneys of pre-disease lupus mice and may contribute to tissue injury by releasing cytotoxic granules (42). Another study showed that SLE patients have an expanded population of CTLs, which may contribute to tissue damage (43). Further studies are needed to determine whether IFN-Is contribute to the activation of these human effector cells.

Persistent IFN-Is exposure, particularly IFN-α, has long been implicated in immune dysfunction and autoimmune diseases, through a number of mechanisms. Some patients treated with IFN-α therapy develop autoimmunity, including RA and lupus-like autoimmune syndrome (44, 45). Chronic IFN-α overexpression *in vivo* induces rapid and lethal lupus, with immune complex glomerulonephritis in NZB/W lupus-prone mice (46). Such excess IFN-α can also induce sustained B cell proliferation *in vivo*, accompanied by uncontrolled production of proliferating, short-lived, autoantibody-secreting plasmablasts in secondary lymphoid organs of NZB/W mice (47). pDC-derived IFN-Is have been shown to increase the translocation of marginal zone B cells to the follicular region of the spleen, which disrupt the ability of marginal zone macrophages to clear apoptotic cells and promote the loss of immune tolerance to apoptotic cell-derived antigens in SLE (48). IFN-Is also promotes affinity maturation of antibodies by activating DCs to produce IL-6 (49). The severity of lupus-related pathology is attenuated with IFNAR-deficiency or IFNAR-blocking antibody in several murine lupus models (50–52).

While the involvement of IFN-Is-IFNAR signaling is a consistent feature of murine lupus models, there is less consensus in RA. In contrast to the association of IFN-α with humoral autoimmunity, IFN-β has homeostatic and anti-inflammatory functions. In RA synovium, IFN-β reduced the secretion of RA-associated proinflammatory mediators, including IL-6, TNF-α, matrix metalloproteinases, and prostaglandin E2 (53). IFN-β also primes an anti-inflammatory phenotype of endothelial cells by upregulating the expression of CD73, an ecto-5′-nucleotidase that produces anti-inflammatory adenosine from AMP, at least in neuroinflammation (54). Other studies have shown that exogenous IFN-β can inhibit autoimmune collagen-induced arthritis (CIA) (55, 56). In contrast, IFN-β deficient mice develop prolonged CIA, with a higher incidence relative to control mice (57). IFN-β delivery has been used therapeutically in multiple sclerosis (58) and has been considered for RA (59). Monoclonal antibody therapies inhibiting IFN-Is signaling or depleting of IFN-overproducing plasmacytoid DCs (pDCs) are under evaluation for the treatment of SLE (60, 61). The opposing roles of IFN-α and IFN-β clearly require careful consideration in relation to these potential IFN-Is-targeted therapies in autoimmunity.

#### TBK1, IFN-Is, and Humoral Autoimmunity

The prevailing concept in SLE and murine lupus models is that immune complexes containing autoantibodies bound to self-DNA and RNA can act as interferogenic stimuli, following Fc receptor-mediated internalization and activation of endosomal TLR7 and TLR9 in pDCs (62). TLR7- or TLR9-mediated induction of IFN-Is, however, does not require TBK1. For instance, TBK1 is not required for IFN-I production in the TLR7-dependent pristane-induced lupus model (51). TLR9 ligand (CpG-B) induces IFN-Is production by B cells and DCs through IRF3, but independently of TBK1. Autocrine IFNAR signaling in B cells is required for enhanced IgM and IgG2a autoantibody production and these are dominant autoantibody isotypes in murine lupus (63).

Using viable TBK1^−/−^ TNF^−/−^ mice, Ishii and colleagues demonstrated functional distinctions between TBK1 signaling in hematopoietic and non-hematopoietic cells for the induction of Ag-specific responses in a plasmid-DNA immunization model (64). TBK1^−/−^ TNF^−/−^ mice had no difference in total serum IgG1 and IgG2a, suggesting normal B cell function. However, TBK1^−/−^ TNF^−/−^ mice had completely abrogated primary and secondary antigen-specific IgG responses upon vaccination with plasmid-DNA, relative to wild type, TNF^−/−^ TBK1^+/+^, Myd88^−/−^, or TRIF^−/−^ mice. Mechanistically, the DNA component of the plasmid-DNA vaccine was shown to activate DCs in a TBK1- and IFN-I-dependent manner, but this occurred independently of the CpG DNA sensor, TLR9 (64). Along the same lines, alum and hydroxypropyl-β-cyclodextrin adjuvants have been shown to induce cell death and DNA release as part of their immunogenic properties and TBK1^−/−^ TNF^−/−^ mice immunized with these adjuvants had reduced levels of antigen-specific IgG1 responses (65, 66). Ishikawa and colleagues subsequently demonstrated that intracellular DNA induced DC activation and IFN-Is production through the cGAS-STING-TBK1 pathway (67).

#### TBK1-Dependent IFN-Is Can Induce Lupus

As mentioned, TBK1-dependent IFN-Is responses are activated by cytoplasmic nucleic acids. In the autoimmune context, pathogenic TBK1-mediated IFN-Is responses can be caused by aberrant self-DNA that leads to chronic IRF3 activation, such as is the case in TREX1 deficiency (68). TREX1 is an endoplasmic reticulum (ER)-associated 3′–5′ exonuclease, which degrades cytoplasmic viral DNA before sensing occurs. TREX1 is also required to clear endogenous retroelements and genomic DNA. TREX1 deficiency in patients and murine models causes lupus-like autoimmune manifestations. TREX1-deficient mice develop aberrant interferogenic responses and features of lupus owing to the cytoplasmic accumulation of endogenous nucleic acids and chronic activation of the TBK1-dependent DNA-sensing pathway (68–70). Mutations in TREX1 are associated with human autoimmune disorders, including Aicardi–Goutières syndrome (71), familial chilblain lupus (72), and SLE (73). An inhibitor of TBK1 was effective in treating TREX1^−/−^ mice (74).

In summary, TBK1 is an important signaling kinase for the induction of IFN-Is in response to a number of ligands that activate TLR3, TLR4, and the STING pathways. TBK1 may be less relevant in other IFN-Is induction pathways, including TLR7 and TLR9. Mutations leading to aberrant activation of TBK1 and IFN-Is overproduction can contribute to lupus. Limiting pathogenic IFN-Is production through TBK1 inhibition may alleviate lupus. However, TBK1-driven responses other than IFN-Is induction may also contribute to humoral autoimmunity and are discussed in the next section.

### TBK1-Regulated Germinal Center (GC) Responses in Humoral Immunity

#### GC-Dependent Humoral Immune Responses

Antibody-mediated autoimmune diseases share underlying immune mechanism(s). High-affinity autoantibodies arise from a GC reaction occurring in the B cell follicles (75, 76). The GC is a specialized structure in secondary lymphoid tissues, where B cells undergo iterative rounds of somatic hypermutation in Ig variable (V) gene segments, class switching and affinity selection, as well as post-translational modifications. Normally, the GC reaction is transient (self-terminating) (77) and only B cells expressing affinity-matured, class-switched antibodies specific for the antigen exit GCs, and survive as long-lived memory B cells and/or antibody-secreting plasma cells (78–80). However, the GC reaction can persist and give rise to antibody-mediated autoimmunity (81).

Intrinsic B cell defects can directly contribute to the development of spontaneous GCs, breakdown of B cell tolerance and humoral autoimmunity, such as *Tlr7* gene duplication (80) or WAS (Wiskott–Aldrich syndrome) protein deficiency (82, 83) in lupus. However, CD4^+^ T_FH_ cells provide another essential cellular component regulating GC B cells. T_FH_ cells are required for the generation of high-affinity antibodies by promoting the GC reaction, including B cell clonal proliferation, affinity selection and the development of high-affinity antibody-producing cells (75, 84, 85). T_FH_ cells are characterized by the expression of chemokine receptor CXCR5, which facilitates migration and proximity to follicular B cells. Here, they provide cognate help to B cells *via* stable interactions such as SAP (SLAM-associated protein), costimulatory molecules such as CD40L, and cytokines such as IL-21, IL-4, and IFN-γ (86–89). While T_FH_ cells are critical for an optimal GC reaction and subsequent generation of protective antibodies following immunization, abnormal development and/or function of T_FH_ have also been implicated in loss of tolerance and the development of humoral autoimmunity.

An increased T_FH_ population in the GC as well as GC numbers may contribute to aberrant positive selection and autoantibody formation in SLE (90). This is exemplified in Sanroque mice, in which exaggerated T_FH_ generation occurs in a cell-intrinsic manner and leads to spontaneous GC formation, and lupus-like pathology (91, 92). Furthermore, adoptively transferred Roquin^san/san^ T_FH_ cells are able to induce spontaneous GC B cell expansion and GC formation in naïve recipient mice (92). Similarly, an enlarged T_FH_ population accompanies increased GC size and more productive humoral responses in immunization models (77). Conversely, mice with conditional Bcl6 deficiency in T cells (Bcl6 is a transcriptional repressor that regulates both T_FH_ and GC B cell differentiation) have impairment of T_FH_ development, GC reactions, and antibody responses (93, 94). CD4 transgenic autoreactive T cells deficient in SAP (SLAM-associated protein, which mediates stable T-B interactions critical for GC formation), failed to mount GC reactions, develop IgG1 autoantibodies, and autoantibody-mediated arthritis (95, 96). Thus, the size of the T_FH_ population is directly coupled with GC function and ensuing humoral responses. Abnormal T_FH_ accumulation may also contribute to the production of pathogenic autoantibodies through enhanced positive selection of self-reactive B cells.

In clinical settings, the frequency of T_FH_-like cells is increased in the peripheral blood of RA patients and correlates with higher elevated levels of anti-CCP (cyclic citrullinated peptide) autoantibodies, as well as disease activity (97, 98). Conversely, treatment responsive, new onset RA patients have a reduced frequency of circulating T_FH_, which is accompanied by a decrease in anti-CCP antibody (98). SLE patients also demonstrate a similar expansion of T_FH_-like cells, which correlates with disease activity, frequency of circulating plasmablasts, and anti-double-stranded DNA antibody positivity (99). T_FH_ expansion and its association with autoantibody responses have also been noted in other humoral autoimmune syndromes, including type 1 diabetes (100) and primary Sjogren’s syndrome (101, 102). Given the robust correlation between T_FH_ numbers and high-affinity autoantibody levels, manipulation of the differentiation program and plasticity of T_FH_ cells may provide new therapeutic options in autoimmune diseases, such as SLE and RA.

#### ICOS in Humoral Immune Responses against TD Antigens and Humoral Autoimmunity

Among many determinants of optimal humoral immunity, ICOS has been consistently associated with GC reactions and the induction of GC-dependent thymus-dependent (TD) antibody responses. ICOS is a critical coreceptor, distinct from CD28, on activated or antigen-experienced T cells (103, 104) and is highly expressed on T_FH_ (105). Through interaction with ICOS ligand (ICOSL) on antigen-presenting cells (DCs and B cells), ICOS delivers robust costimulatory signals that promote T_FH_ positioning and thus supports GC function (106). ICOSL^−/−^ mice mount comparable antigen-specific IgM and IgG3 responses, but have reduced IgG1 and IgG2a production upon immunization with thymus-independent antigens (107). ICOS^−/−^ or ICOSL^−/−^ mice have defective production of class-switched antibodies against TD antigens (particularly IgG1, IgG2a, and IgG2b isotypes, but not IgM), along with reduced number and size of GCs and a lack of B cell memory (107–110). Additionally, mice with a tyrosine-to-phenylalanine point mutation at residue 181 in the cytoplasmic tail of ICOS have abrogated T_FH_ generation, GC reactions, antibody class switching, and antibody affinity maturation (111). ICOS deficiency or antibody-mediated depletion of ICOS-expressing CD4^+^ T cells in SLE1 lupus mice results in diminished pathogenic T_FH_ expansion, inhibited plasma cell generation, and a reduction in class-switched IgG autoantibodies (112). ICOSL^−/−^ or B cell-specific ICOSL^−/−^ mice have markedly inhibited development of proteoglycan-induced arthritis, with notable reductions in T_FH_ and GC B cells, IL-21 production, and proteoglycan-specific IgG antibody responses (113).

Blockade of the ICOSL pathway ameliorates autoimmune CIA, the K/BxN spontaneous arthritis model, and the SLE (NZB/NZW) F1 mouse model, with marked reductions in disease manifestations, numbers of T_FH_ and GC B cells, and pathogenic, class-switched, high-affinity autoantibodies (113–115). Interestingly, inhibition of CIA was observed even when ICOSL blocking antibody was given after the onset of disease (114). Sanroque mice have excessive ICOS activation due to genetic mutation of a RING-type ligase that represses ICOS (91). These mice develop spontaneous GC in the absence of foreign antigen, increased numbers of T_FH_ cells, spontaneous autoantibodies, including antinuclear antibodies, and lupus-like manifestations, such as glomerulonephritis. Intriguingly, although ICOS or ICOSL deficiency in Sanroque mice substantially reduced autoantibody production, it did not result in complete inhibition of autoantibody production (91). This observation suggests a contribution from residual GC-independent extra-follicular pathway (85, 113). Thus, ICOS/ICOSL signaling drives optimal GC-dependent TD antibody responses and inhibition of this pathway abrogates the GC reaction, autoantibody responses, and disease features in humorally mediated autoimmune disease models. Indeed, therapies targeting ICOS/ICOSL are under evaluation in early phase clinical trials of SLE (116).

#### TBK1 Mediates ICOS Signaling for T_FH_ Maturation and GC-Mediated Antibody Responses

Similar to other CD4^+^ T cell subsets (Th1, Th2, Th17, and Treg cells), T_FH_ development is a multi-step process which involves initial priming of naïve CD4^+^ T cells by dendritic cells in the T cell zone, followed by expansion and differentiation that are regulated through signaling pathways activated downstream of cytokines and cell surface molecules. Subsequent activation of lineage-defining transcription factors (T-bet for Th1, GATA3 for Th2, RORγt for Th17, FoxP3 for Treg cells, and Bcl6 for T_FH_) promotes T cell differentiation (93, 94, 117).

T follicular B helper development can be separated into two stages—(i) naive to Bcl6^+^ pre-T_FH_ and (ii) pre-T_FH_ to mature GC T_FH_. Pre-T_FH_ development follows DC priming *in vivo*, through an ICOS costimulation signal and the phosphoinositide-3 kinase (PI3K) pathway. The ICOS-PI3K pathway instructs T_FH_ differentiation *via* induction of Bcl6 and the subsequent Bcl6-dependent expression of CXCR5 on pre-T_FH_ (23, 105). The activation ICOS-PI3K signaling alone is, however, insufficient to drive full GC T_FH_ maturation and the GC reaction (23, 111, 118). For final differentiation of nascent T_FH_ into GC T_FH_, pre-T_FH_ cells require a second costimulatory signal through ICOS (119, 120). This has been demonstrated by the inability of T cell-selective deletion of PI3K components to fully recapitulate the phenotype of CD4^+^ T cells from ICOS^−/−^ mice (111, 121).

A recent report identified TBK1 as a unique signaling kinase in the ICOS pathway (23). In this study, Pedros and colleagues identified a conserved TRAF-like motif in the cytoplasmic tail of ICOS (iProx motif), which mediated TBK1 recruitment and activation following a combination of strong TCR and ICOS signals. These authors showed that by deleting the iProx motif on ICOS specifically in CD4^+^ T cells, TBK1 failed to associate with ICOS. T cells modified in this way displayed severely impaired differentiation into GC T_FH_ and TD antibody responses, despite generating pre-T_FH_ cells. A similar effect was obtained by CD4^+^ T cell-specific TBK1 depletion. In a series of reconstitution experiments, transducing ICOS and TBK1 constructs into ICOS^−/−^ TCR transgenic CD4^+^ T cells, it was shown that intact ICOS is required for the generation of both nascent and final GC T_FH_ populations, while TBK1 controls progression from the pre-T_FH_ to mature GC T_FH_ phenotype (23).

Although the downstream mediators of ICOS-TBK1 signaling in T_FH_ have not been identified, FoxO1 is a potential candidate because ICOS signaling instructs the T_FH_ program *via* AKT-mediated FoxO1 phosphorylation (119). AKT has been shown to be a TBK1 substrate in some settings (13, 14, 122). FoxO1 is a transcription factor that, in its active unphosphorylated state, represses T_FH_ programming. FoxO1 phosphorylation results in its transient inactivation and cytoplasmic translocation from the nucleus (119). FoxO1 inactivation also reduces FoxO1-dependent KLF2 expression, together with expression of KLF2-dependent chemokine receptors, necessary for optimal repositioning of T_FH_ in the GC (120). FoxO1 inactivation, specifically in CD4^+^ T cells (*Foxo1*^fl/fl^:CD4-Cre mice) caused defective Tregs and systemic autoimmunity, characterized by accumulation of the T_FH_ population, with exaggerated Bcl6 induction and GC formation, and production of anti-DNA antibodies (123). While FoxO1-sufficient CD4^+^ T cells give rise to effector T cells, pre-T_FH_, and GC T_FH_ upon immunization, FoxO1^−/−^ CD4^+^ T cells generate pre-T_FH_ cells with higher expression of T_FH_-defining markers (Bcl6, CXCR5, and PD-1) and lowered T cell zone chemokine receptors (CD62L, PSGL1) (119). ICOS-driven FoxO1 inactivation thus alters the chemokine receptor profile of pre-T_FH_, facilitating migration from the T cell zone toward the B cell follicles (119). Conversely, ICOS/ICOSL blockade results in the relocation of fully developed T_FH_ back to the T cell zone. This relocation reverses their phenotype toward non-T_FH_ effector T cells, with a consequent reduction in antigen-specific GC B cells, as well as serum antigen-specific IgG1 and IgG2a responses, indicating collapse of the GC response (120). This study also concluded that ICOS is not required for T_FH_ survival or expression of T_FH_-related transcription factors, but rather, it regulates the expression of T_FH_ homing markers. Changes in T_FH_ transcription factors are thus likely to be a secondary effect upon failure to maintain the positioning of pre-T_FH_ and impaired costimulatory signals from follicular B cells (120).

Although TBK1 has been identified as an ICOS-specific signaling kinase required for full maturation of GC T_FH_, the role of TBK1-mediated FoxO1 regulation in this process has not been elucidated. One study using conditional TBK1^−/−^ in CD4^+^ T cells (*Tbk1*^fl/fl^:CD4-Cre mice) and stimulation with TCR and CD28, suggested that basal TBK1 is required for constitutive AKT turnover to prevent hyperactivation of AKT upon T cell activation (124). These authors also reported a marked increase in IFN-γ production and activation markers in CD4^+^ T cells derived from *Tbk1*^fl/fl^:CD4-Cre mice, indicating the propensity of these cells to become a Th1-like population in the absence of basal TBK1 (124). Interestingly, ICOS^−/−^ or ICOSL^−/−^ mice also exhibit enhanced Th1 responses in secondary lymphoid tissues with marked elevation of IFN-γ in the context of infection (125–127).

Given the role of TBK1 in ICOS signaling in GC T_FH_ and downstream GC-driven antibody responses, TBK1 inhibition may curtail humoral autoimmunity through an ICOS-driven GC pathway. Importantly, targeting ICOS/ICOSL and/or TBK1 may not result in generalized immunosuppression, but rather reverse cell fate decisions in T_FH_. Understanding how TBK1 signals in T_FH_, how it affects cell fate decisions in T helper cell polarization, positioning and migratory pathways may provide new therapeutic strategies, especially for antibody-mediated autoimmunity.

### TBK1-Regulated Autophagy in Immune Regulation

#### TBK1 Regulates Autophagy

The functional effects of TBK1 extend beyond innate immune signaling. Autophagy is a conserved homeostatic process in eukaryotic cells involving sequestration and lysosomal degradation of cytoplasmic contents, including damaged or surplus organelles (mitophagy for mitochondria, pexophagy for peroxisomes, ribophagy for ribosomes, reticulophagy for endoplasmic reticulum), cytotoxic macromolecular aggregates (aggrephagy), and intracellular microorganisms (xenophagy) (128–131). Autophagy will not be discussed in detail as it has been extensively reviewed elsewhere (128–131). Instead, we discuss reports which have implicated TBK1 in autophagic processes and how these translate to immune responses.

#### TBK1 in Antimicrobial Autophagy (Xenophagy)

TANK-binding kinase 1-mediated regulation of autophagy has been described in the context of antimicrobial defense (xenophagy), in which intracytoplasmic pathogens are sequestered into autophagosomes and targeted for lytic, lysosomal degradation. TBK1 and its homolog IKKε have been identified as binding partners of the autophagy receptor protein NDP52 that recognizes polyubiquitylated *Salmonella enterica* in human cells. However, only TBK1 is required for xenophagy of *S. enterica* (132, 133) and mycobacteria (134). Canonical IKKs initiate autophagy, while TBK1 knockdown suppresses the maturation of autophagosomes into autolysosomes. Mechanistically, TBK1 phosphorylates autophagy receptor proteins, including NDP52, OPTN on Ser177, and p62 (also known as SQSTM1) at Ser403 (located at the ubiquitin-associated/UBA domain) (132–134). Phosphorylation increases the affinity of LC3-binding autophagy adaptors for K48- and K63-ubiquitinated cytoplasmic bacteria, as well as polyubiquitinated protein aggregates (135), and it promotes autophagic clearance (132–134). Knockdown or pharmacological inhibition of TBK1 impairs autophagic killing of *S. enterica* or *M. tuberculosis* (133, 134). Mice deficient in autophagic proteins (Atg3, Atg5, Atg7, Atg9, and Atg16L1) have embryonic lethality (129). Viable conditional autophagy knockout mice often have impaired pathogen clearance, reduced survival, and severe tissue injury due to enhanced inflammasome and cytokine responses, and in some cases, enhanced Th17 responses (136–138). In the same study, TBK1 was shown to be important for delivery of the lysosomal hydrolase cathepsin D to the autophagolysosomal compartment (134). TBK1, therefore, appears to play an essential role in late autophagic flux.

#### TBK1 in Mitophagy and Potential Implications in Neuronal Health

Autophagy is increasingly appreciated for its role in maintaining cell homeostasis through clearance or normal turnover of cytoplasmic contents or defective cellular organelles, including mitochondria (mitophagy). Mice deficient for Atg5 specifically in neural cells develop progressive decline in motor function in the absence of any disease-associated mutant proteins, accompanied by the accumulation of cytoplasmic inclusion bodies in neurons (139). Damaged mitochondria are detrimental to cellular homeostasis and efficient removal through autophagy is crucial for cell survival, particularly for senescent cells, such as neurons, which cannot dilute cytotoxic contents through cell division (140). Mitochondrial damage induces concomitant PINK1-PARKIN-mediated poly-ubiquitylation of damaged mitochondria and also activates TBK1. In turn, TBK1 can phosphorylate autophagy receptors (OPTN, SQSTM1/p62, and NDP52), thereby enhancing the ability of these receptors to associate with ubiquitinated cargo (e.g., ubiquitin-tagged, depolarized mitochondria) and autophagic membranes (24, 25, 28, 141). This post-translational modification creates a signal amplification loop that recruits and retains autophagy receptor/TBK1/ubiquitinated cargo complexes, thereby promoting mitophagy. Separate mutations that disrupt TBK1’s association with OPTN, or of OPTN with ubiquitin, abolish the translocation and activation of TBK1, and, therefore, impair mitophagy (25).

Exome sequencing identifies TBK1 as a neurodegenerative disease gene in amyotropic lateral sclerosis (ALS) and frontotemporal dementia (26, 27). Further, it was shown that mutations of TBK1 at the C-terminal TBK1 coiled-coil domain, resulted in TBK1’s dissociation from OPTN, while preserving its kinase activity (located at the N-terminal ubiquitin-like domain) (27). These studies provide a potential mechanistic basis for TBK’s involvement in ALS. Mutations linking OPTN to impaired autophagy and neurodegenerative diseases have also been characterized (142). Thus, although it has not been directly demonstrated, TBK1-regulated autophagy appears to maintain cellular homeostasis of long-lived neuronal cells through mitophagy.

#### TBK1 and Autophagy in Immune Cell Lineage Development

TANK-binding kinase 1-regulated autophagy may also be important for regulation of immune cells. As mentioned above, mice deficient in autophagic proteins (Atg3, Atg5, Atg7, Atg9, and Atg16L1) have neonatal lethality, as do TBK1^−/−^ mice (1, 129). In contrast, STING^−/−^ mice are viable, but have impaired TBK1-dependent IFN-I responses to cytoplasmic DNA (67). Autophagy allows dynamic changes necessary for proper mammalian development through the recycling and provision of macromolecules and clearance of apoptotic bodies. Conditional *Atg7*^fl/fl^:Vav-Cre mice (i.e., hematopoietic cell-specific deletion of Atg7) overcomes embryonic lethality, but these mice are anemic and lymphopenic, linking autophagy to erythropoiesis and lymphopoiesis (129). Similarly, T cell-specific deletion of Atg5 or Atg7 (*Atg5*^fl/fl^:Lck-Cre or *Atg7*^fl/fl^:Lck-Cre mice) or innate lymphoid cell (ILC)-specific deletion of Atg5 (*Atg5*^fl/fl^:Nkp46-Cre) reduces peripheral T cells and ILC subpopulations, respectively (143, 144). Maturation of naïve T cells depends on autophagy to reduce mitochondrial and ER contents through mitophagy and reticulophagy, respectively (143, 145, 146). Defective autophagy in T cells results in accumulated mitochondrial biomass, disturbed Ca^2+^ homeostasis, higher levels of reactive oxygen species (superoxide), and enhanced susceptibility to apoptosis (143, 145, 146). In contrast to lymphopenia, the myeloid compartment in *Atg7*^fl/fl^:Vav-Cre mice is expanded (147), implicating autophagy in the balance between lymphopoiesis and myelopoiesis. It was also recently shown that autophagy is required for full granulopoiesis (148). Autophagy regulates cellular differentiation and activation by accommodating metabolic adaptation, which can occur in parallel and independently of transcriptional regulators. Neutrophils from *Atg7*^fl/fl^:Vav-Cre or *Atg7*^fl/fl^:Cebpa-Cre (granulocyte-macrophage progenitor-specific deletion of Atg7) mice are numerically expanded, but are unable to complete maturation and, therefore, are functionally defective. In this case, autophagy-mediated lipolysis (lipophagy) provides free fatty acids to support a mitochondrial respiration pathway essential for neutrophil differentiation (148). Autophagy regulates cytosolic processing of antigen for presentation on MHCII in DCs. The absence of Atg5 in DCs results in failure to mount full Th1 cell immunity to viral infection (149). TBK1 has also been associated with metabolic adaptation of DC after TLR stimulation, whereby TBK1 phosphorylates AKT for the glycolysis which is necessary for DC activation. shRNA-mediated TBK1 knockdown in DCs results in a blunted glycolytic shift and reduced ability of these DCs to prime antigen-specific T cells *in vitro* (14).

In summary, separate lines of evidence have linked autophagy to cell metabolism, TBK1 to autophagy, and TBK1 to metabolism in immune cell development and activation. Further studies of TBK1’s role in cellular autophagy and metabolism in various immune contexts could allow manipulation of immune function—either for protection against pathogens or rewiring toward tolerance in autoimmunity.

#### TBK1 and Autophagy Balance Age-Related Inflammation

In contrast to the maturation defect of neutrophils in the absence of autophagy, macrophages derived from *Atg5*^fl/fl^:LysM-Cre mice display a heightened proinflammatory phenotype. These mice develop greater hepatitis on a high fat diet and low dose LPS and also spontaneous uveitis (150, 151). Autophagy-deficient macrophages activate the NLRP3 inflammasome and develop IL-1β-mediated inflammation (151–153). Similar to macrophages, Atg16L1-deficient DCs have heightened activation in graft-versus-host disease (154). Loss of function polymorphisms of Atg16L1 have been associated with age-dependent development of inflammatory bowel disease (Crohn’s disease) owing to impaired clearance of ileal pathogens or endogenous protein aggregates, and chronic elevation of inflammatory cytokine responses (155, 156). Interestingly, DC-specific deletion of TBK1 (*Tbk1*^fl/fl^:CD11c-Cre mice) also display age-related cellular hyperactivation, with marked upregulation of costimulatory molecules on DCs, T cell activation, and autoimmune features (splenomegaly, lymphadenopathy, and tissue infiltration with lymphocytes) (157). These *Tbk1*^fl/fl^:CD11c-Cre mice have an increased frequency of activated IFN-γ-producing CD4^+^ and CD8^+^ T cells, while Tregs remain comparable to TBK1-sufficient mice. Consequently, these mice are more sensitive to EAE and mount more robust antitumor immunity against poorly immunogenic B16F10 melanoma cells (157).

Enhanced macrophage or DC activation in the absence of TBK1 may be due to impaired autophagy, which normally limits age-related inflammasome activation. Of note, the age-dependent hyperinflammatory status of autophagy- or TBK1-deficient macrophages and DCs in Atg conditional knockout mouse models resembles aging macrophages. These cells shift from an anti-inflammatory to a proinflammatory phenotype, with an age-related reduction in autophagic activity and sensing of endogenous damage-associated molecular patterns (DAMPs) (158, 159). Thus, it is tempting to speculate that TBK1 maintains cellular autophagy and sustains immune cell longevity and homeostasis. Hallmarks of accelerated immune cell aging with chronic TBK1 deficiency are also notable in the *Tbk1*^fl/fl^:CD19-Cre mice (B cell-specific ablation of TBK1) (160). These mice have normal B cell populations in the spleen and bone marrow, but develop age-related dysregulation of the non-canonical NF-κB pathway, uncontrolled production of IgA, increased levels of autoantibody antinuclear antigen and anti-dsDNA, with nephropathy-like disease (160). In this study, it was concluded that steady state TBK1 negatively regulates IgA class switching in B cells by attenuating noncanonical NF-κB signaling. This effect was thought to be due to TBK1-mediated phosphorylation and degradation of NF-κB-inducing kinase, downstream of BAFF or APRIL signaling (160). Intriguingly, it is possible that *Tbk1*^fl/fl^:CD19-Cre mice phenocopy the aging B cell repertoire because the B1 population (responsible for IgA responses) and autoantibody production are enhanced by age (161). While chronic deficiency of autophagy or TBK1 results in amplified endogenous inflammation to DAMPs in myeloid cells and abnormalities in the B cell repertoire, inhibition of autophagy may be exploited to target long-lived autoimmune populations (discussed below) through acceleration of immune cell aging.

#### Autophagy Supports Long-Lived Memory Immune Cells—Implication of TBK1

As discussed above, autophagy is cytoprotective in senescent cells, such as neurons. This cytoprotective function also appears to apply in long-lived immune cells. Mice with B cell-specific deletion of Atg5 or Atg7 (*Atg5*^fl/fl^:CD19-Cre or *Atg7*^fl/fl^:CD19-Cre mice) have mostly normal B cell development, but are unable to maintain long-lived humoral antibody responses owing to the failure to maintain long-lived plasma cells and memory B cells, respectively (162, 163). In both studies, plasma cells and memory B cells arising from immunization and GC were shown to upregulate components of the autophagic machinery. In plasma cells, autophagy is thought to maintain longevity by reticulophagy (autophagic clearance of redundant endoplasmic reticulum) to limit excessive antibody synthesis and conserve energy balance (162). Further studies are needed to investigate whether TBK1 is involved in reticulophagy and physiological adaptation of plasma cells.

Autophagy also supports the lifespan of quiescent, antigen-experienced, long-lived, GC-derived memory B cells through mitophagy. *Atg7*^fl/fl^:CD19-Cre mice mount normal primary antibody responses and have normal differentiation of post-GC memory B cells, but fail to generate secondary antibody responses to influenza virus due to spontaneous death of memory B cells (163). Memory B cells from *Atg7*^fl/fl^:CD19-Cre mice are unable to efficiently remove damaged mitochondria, resulting in accumulation of reactive oxygen species, lipid peroxidation, and oxidative stress-induced death. Interestingly, these mice also develop enhanced Th17 responses to viral infection, possibly as a compensatory mechanism (163). Whether TBK1 also regulates mitophagy and, therefore, the survival of memory B cells requires further investigation. Mitophagy was shown to support the generation of LCMV-induced memory CD8^+^ T cells (164, 165) and MCMV-induced memory NK cells (166). These studies highlight that autophagy is not apparently required for germline T cell development, nor for T cell activation and proliferation, but is important for established effector T cells to generate a pool of memory cells (165, 166). Autophagy is dynamically induced at various stages of immune cell development, activation, and differentiation, but plays a particular role in the formation and maintenance of long-lived immune populations, including memory B cells and senescent plasma cells. Because long-lived plasma cells or plasma cells deriving from memory B cells can drive persistent autoimmune disease (167), abrogation of autophagy through TBK1 inhibition might reduce resistance to autonomous cell aging and death, and diminish pathogenic autoantibody responses.

In summary, a number of studies demonstrate significant overlap between TBK1 and autophagy, most notably in antimicrobial xenophagy and maintenance of neuronal cell health. Similar to autophagy, TBK1 has a complex role in immune cells. It is known that autophagy is dynamically regulated to accommodate rapid metabolic adaptation and organelle turnover associated with cell development, differentiation, activation, and longevity. TBK1 regulates autophagy through post-translational modifications of autophagy adaptor/receptor proteins required for the maturation of autophagosomes. TBK1’s involvement in other types of organelle autophagy and metabolic signaling pathways in immune lineage cells and immune responses is of great interest. Autophagy supports the extended lifespan of cells, such as neurons, immune memory populations, and long-lived plasma cells. Targeting autophagy through the inhibition of TBK1 may provide a novel approach for treating humoral autoimmune diseases. Inhibition of autophagy or TBK1 may favor the generation of short-lived effector cells, rather than long-lived memory populations. Both autophagy and TBK1 have been shown to regulate the delicate balance between cellular adaptation for efficient immune response and aging-associated autoinflammation. Studies exploring how, when, and where TBK1 facilitates autophagy in distinct immune lineages will inform potential modulation of protective or pathogenic immune responses.

## Conclusion

In this review, we discuss the remarkable functional diversity of TBK1 in the context of humoral autoimmunity. These pathways are summarized in the Figure 1. TBK1 is required for IFN-Is production in the context of sensing viral or aberrant cytoplasmic nucleic acids. Overactive TBK1 can precipitate IFN-Is and lupus, such as is the case for TREX1 deficiency. Recent literature reports that TBK1 is associated with humoral antibody responses *via* its recruitment to and activation of ICOS in the CD4^+^ T_FH_ population in GCs of lymph nodes. ICOS is required for full maturation of the GC T_FH_ population, the GC reaction, GC-mediated generation of affinity-matured long-lived plasma cell and memory B cells, and productive GC-derived antibody responses. Thus, TBK1 inhibition may be useful in pathogenic autoantibody responses mediated by GC. Finally, we highlight the similarity of TBK1 deficiency to that of autophagy-deficiency. Therapeutic TBK1 inhibition may therefore lead to premature aging and/or death of pathogenic immune cells, such as long-lived plasma cells, and memory B cells in autoimmune diseases.

## Author Contributions

All authors contributed to the assembly and revision of this review manuscript.

## Conflict of Interest Statement

The authors declare that the research was conducted in the absence of any commercial or financial relationships that could be construed as a potential conflict of interest.
